# Kinetin induces cell death in root cortex cells of *Vicia faba* ssp. *minor* seedlings

**DOI:** 10.1007/s00709-012-0466-7

**Published:** 2012-11-11

**Authors:** Anita Kunikowska, Anna Byczkowska, Andrzej Kaźmierczak

**Affiliations:** Department of Cytophysiology, Faculty of Biology and Environmental Protection, University of Łódź, Pomorska 141/143, 90236 Łódź, Poland

**Keywords:** Cell cycle, Cell death, Cell dehydrogenases, Fluorescence, Reactive oxygen species, Calcium ions

## Abstract

The double fluorescence staining with acridine orange and ethidium bromide (AO/EB) revealed that treatment of *Vicia faba* ssp. *minor* seedlings with kinetin-induced programmed cell death (PCD) in root cortex cells. Kinetin-induced cell death reflected by the morphological changes of nuclei including their invagination, volume increase, chromatin condensation and degradation as well as formation of micronuclei showed by AO/EB and 4,6-diamidino-2-phenylindol staining was accompanied by changes including increase in conductivity of cell electrolytes secreted to culture media, decrease in the number of the G1- and G2-phase cells and appearance of fraction of hypoploid cells as the effect of DNA degradation without ladder formation. Decrease in the number of mitochondria and in the activity of cellular dehydrogenases, production of reactive oxygen species (ROS), appearance of small and then large lytic vacuoles and increase in the amount of cytosolic calcium ions were also observed. The PCD was also manifested by increased width and weight of apical fragments of roots as well as decreased length of cortex cells which led to shortening of the whole roots. The kinetin-induced PCD process was almost completely inhibited by adenine, an inhibitor of phosphoribosyl transferase, and mannitol, an inhibitor of ROS production. These cell-death hallmarks and pathway of this process suggested that the induction of kinetin-specific vacuolar type of death, expressed itself with similar intensity on both morphological and metabolic levels, was a transient protecting whole roots and whole seedlings against elimination.

## Introduction

Development of organisms depends on many physiological processes, including cell division and programmed cell death (PCD), which control proper growth of multicellular (van Doorn and Woltering 2005; Delaval and Birnbaum 2007; Hübner et al. 2009) as well as unicellular organisms (Shemarova 2010). Cell division, elevating the number of cells (Delaval and Birnbaum 2007) as well as PCD, eliminating physiologically redundant, damaged or abnormal cells (van Doorn and Woltering 2005) plays an important role in the wide range of differentiation processes (Barciszewski et al. 2007).

In animals, cell death proceeds through apoptosis, micro- and macroautophagy and non-lysosomal type of death and necrosis (van Doorn and Woltering 2005) as well as via mitotic catastrophe (Hübner et al. 2009; McCall 2010). In plants, cells can die via vacuolar and necrotic as well as mixed type of death but not via apoptosis (van Doorn et al. 2011). However, some of the morphological and metabolical features of animal and plant cell death, regardless of its type, are similar (van Doorn and Woltering 2005; Collazo et al. 2006; Jan et al. 2008; van Doorn et al. 2011).

PCD can be induced via internal as well as external signals including environmental cues (van Doorn and Woltering 2005) as well as many chemical agents (Rao et al. 2008). Plants produce numerous substances (Taraphdar et al. 2001; Rao et al. 2008) which are widely studied with respect to anticancer therapy (Taraphdar et al. 2001; Doležal et al. 2007; Rao et al. 2008). There are phenols and phenolic acids, polyphenolic flavonoids, sugars, glicoproteins, lignins, alkaloids (Taraphdar et al. 2001; Rao et al. 2008) as well as cytokinins, known plant and animal growth regulators (Barciszewski et al. 2007), which can induce PCD in human and animal as well as in plant cells (Carimi et al. 2003; Choi et al. 2008; Doležal et al. 2007; Mlejnek et al. 2003). In human and animals, kinetin riboside, isopentenyladenosine and benzylaminopurine riboside inhibit growth and promote apoptosis prior to cell differentiation process (Ishii et al. 2002). Kinetin ribosides induce apoptosis and suppress HeLa and mouse melanoma (B16F-10) cell growth through the classical mitochondrium-dependent pathway, including disruption of the mitochondrial membrane potential, releasing cytochrome c, activation of caspase-3 and up- and down-regulation of Bcl-2 and Bad proteins. However, human skin fibroblast CCL-116 and bovine primary fibroblast cells are resistant thus no significant changes in Bad, Bcl-XL and cleavage of PARP were observed (Choi et al. 2008). It was demonstrated that kinetin ribosides showed very strong cytotoxic activity against various cancer cell lines and non-cytotoxic activity towards the normal murine fibroblast cell line (NIH/3 T3; Doležal et al. 2007). It is worth noting that kinetin ribosides are more effective anticancer agents then other cytokinins (Griffaut et al. 2004; Doležal et al. 2007).

It was reported that naturally occurring plant cytokinins, kinetin and zeatin or 6-benzylaminopurine (BAP), did not trigger tumour death. They are not active against human M4 Beu and murine B16 melanoma cells (Griffaut et al. 2004), myeloid leukaemia HL-60 cells and human epidermal keratinocytes (Ishii et al. 2002; Berge et al. 2006). However, zeatin and BAP can induce PCD in plants (Carimi et al. 2003). BAP, at 13- and 27-μM concentrations, induced PCD in both carrot (*Daucus carota* L.) and *Arabidopsis thaliana* (L.) Heynh cell cultures, respectively, accelerating senescence of leaves, causing their yellowing with PCD hallmarks including chromatin condensation, oligonucleosomal DNA degradation (laddering), cytochrome c release and inhibition of cell proliferation (Carimi et al. 2003). BAP induced PCD in cells of epidermal and sub-epidermal layers in cotyledons of *Lycopersicon esculentum* and *Solanum aviculare* (Gahan et al. 2003), and its hallmarks were similar to those observed during apoptosis in mammalian, insect and nematode species (Gahan et al. 2003). BAP can also inhibit the PCD process. Such an inhibitory effect was observed in *Nicotiana suaveolens* × *Nicotiana tabacum* hybrid cells at high levels (0.8, 4.0 or 20 mM) of BAP. However, 0.04 μM of BAP at 28 °C induced changes similar to apoptosis suppressing the percentage of dead cells and extending nuclear fragmentation. In the hybrid cells, at higher levels of BAP, positive terminal deoxynucleotidyl transferase-mediated dUTP nick end labeling (TUNEL) signals and accumulation of formazan, indicating production of reactive oxygen species (ROS), were detected less frequently than at its lower levels (Kobori et al. 2007). However, application of TUNEL method to study cell death would not be an unequivocal test because it shows DNA breaks which are not necessarily related to the studied processes (Kobori et al. 2007).

Kinetin naturally occurring in human, animals and plants (Barciszewski et al. 2007), which does not induce cell death in human and animal cells (Berge et al. 2006; Ishii et al. 2002), has not been studied in plants so far. Fluorescence staining with acridine orange/ethidium bromide (AO/EB) allowing to express the level of cell death as a cell death index together with 4, 6-diamidino-2-phenylindol (DAPI) staining showed morphological changes in nuclei and nuclear chromatin, indicating that kinetin acted as an inducer of programmed death in root cortex parenchyma cells of *Vicia faba* ssp. *minor* seedlings. Kinetin-induced PCD process accompanied with changes in the number of cells in G1 and G2 phases of the cell cycle, in the activity of cellular dehydrogenases, in the ROS production, amount of cytosolic calcium ions, conductivity of cell electrolytes secreted from roots to the culture media and in the morphology of cells and roots was almost completely inhibited by adenine, an inhibitor of phosphorybosyl transferase (Mlejnek and Doležel 2005), and mannitol, the ROS scavenger (Jennings et al. 1998).

## Material and methods

### Plant material, treatment and analyses

Roots of 3-day-old *V*. *faba* ssp. *minor* seedlings treated with respective agents, were used in the studies which were carried out to show the most important hallmarks of PCD induced by 46.0-μM concentration of kinetin (Sigma) and mechanism of its induction using adenine (50 μM; Sigma) and mannitol (50 μM; POCH) with or without kinetin.

**Fig. 1:**
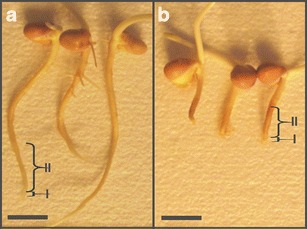
Control (**a**) and kinetin-treated (**b**) seedlings of *V*. *faba* ssp. *minor*. *Scale bars* are 20 mm

To show hallmarks of kinetin-induced PCD, (1) length of seedling roots, (2) weight and (3) width of 2-cm long apical fragments, (4) conductivity in the culture media using the conductivity meter (Elmetron, Poland) as well as (5) cell lengths were measured. Moreover, (6) estimation of DNA content and the number of the cells in phases of the cell division cycle after DAPI staining, (7) measurement of the activity of cellular dehydrogenases with 3-(4,5-dimethylthiazol-2-yl)-2,5-diphenyltetrazolium bromide (MTT; Sigma), (8) determination of the distribution and the amount of calcium ions after chlortetracycline (CTC; Sigma) staining, (9) detection of ROS with nitroblue tetrazolium (NBT; Sigma) and (10) the effect of adenine and mannitol on kinetin-induced cell death were carried out. To detect cell death, (11) the staining with acridine orange (Sigma) and ethidium bromide (Serva) was carried out while (12) morphology of nuclei and nuclear chromatin was examined after AO/EB and DAPI (Sigma) staining. Some of the analyses (6, 7, 11 and 12) were done in two zones of roots (Fig. 1a, b; zone I containing meristem cells and zone II containing growing and differentiating cells).

The fluorescence and white light observations were carried out with Optiphot-2 epi-fluorescence microscope (Nikon) equipped with a UV (UV2A) and blue light (B2A) filter, DDX camera and Act-1 (Precoptic, Poland) software (Kaźmierczak 2010).

### *In planta* cell death estimation

Non-fixed, 2-cm long apical fragments of roots treated with kinetin for 48, 72 and 96 h were cut from the seedlings, washed two times with 0.01 M Na^.^phosphate buffer at pH 7.4 (PHB) and next stained with the mixture of 100 μg^.^ml^−1^ AO and 100 μg^.^ml^−1^ EB at PHB for 5 min. Then, the fragments were washed two times with PHB and fixed with 1 % glutaraldehyde (Merck) at PHB for 15 min, cut along their long axes into very thin sections, washed three times with PHB, observed and photographed using fluorescence microscope (Byczkowska et al. 2012). The changing colour of chromatin, from green to red, allowed to distinguish living, dying and dead cells after the measurement of resultant fluorescence intensity (RFI) of green AO, migrating into the nuclei via cell membrane which did not change its permeability or integrity, and red EB, permeating to dying or dead cells in which cell membrane permeability or integrity was changed (Ribble et al. 2005; Kobori et al. 2007). The RFI values increased with the changing colour from green to red (Byczkowska et al. 2012). This staining also showed changes in the morphology of nuclei and nuclear chromatin. About 350–400 nuclei in each of three experimental series were analysed.

### DAPI staining and determination of DNA content

The 2-cm-long apical fragments of roots of the untreated or 72-h kinetin-treated seedlings were fixed in cold Carnoy’s (96 % ethanol and glacial acetic acid; 3:1) for 1 h, washed with 96 and 70 % ethanol and hydrated. Then, these fragments were stained with DAPI according to the following procedure: 5-min pretreatment with 0.2 M citric acid and 0.1 % Tween; 5-min staining with DAPI (2 μg ml^−1^) together with 0.1 M Na_2_HPO_4_ and 0.2 M citric acid in 9:1 ratio; 5-min washing with the mixture of Na_2_HPO_4_ and citric acid (Hotz et al. 1992). After this procedure, the roots were cut along their long axes into thin sections, washed three times with PHB, analysed and photographed under UV light of fluorescence microscope (Kaźmierczak 2010). The microphotographs were used to measurement of DNA content using the Scn Image software. DNA content of about 450–550 nuclei in each of three experimental series was used to prepare the histograms and to determine the number of cells at particular stages of cell division cycle. The microphotographs were also used to present the morphological changes in nuclei and nuclear chromatin of dying cells.

### Determination of cellular dehydrogenase activity

Activity of dehydrogenases secreted from 2-cm-long apical parts of roots of the untreated and 72-h kinetin-treated seedlings were measured with MTT. Fragments of roots, divided into zone I and zone II (Fig. 1a, b), were washed with 0.01 M Na^.^phosphate saline buffer (PBS) and then placed in 0.9 ml of PBS buffer with 100 μl of 0.5 mg^.^ml^−1^ MTT for 2 h. Next, the 375 μl of the reaction mixture were added with 1,125 μl of acidified isopropanol, and absorbance at 570 nm was spectrophotometrically (JenaMed) measured. Dehydrogenases activity expressed in U (unit) were calculated as the amount of blue formazan produced per 1 min of 1 g of fresh weight of 2-cm-long apical part of roots.

### Determination of ROS production, analyses of the amount and distribution of calcium ions and vacuole detection

Reactive oxygen species were determined in two root zones (Fig. 1a, b) with 0.05 % NBT in PHB. The 2-cm-long living apical fragments of roots of the untreated and 48-, 72- and 96-h kinetin-treated seedlings were washed with PHB and stained in dark for 1 h. Next, these fragments were fixed with 2.5 % glutaraldehyde in PHB for 15 min, then they were washed two times with PHB, analysed and photographed under the white light of microscope.

Calcium ion determinations were carried out in the apical part of roots of the untreated and 48-, 72- and 96-h kinetin-treated seedlings fixed with 2.5 % glutaraldehyde at PHB for 10 min. The plant material was washed three times for 5 min with 50 mM Tris–HCl pH 7.45 buffer (THB), stained for 5 min with 100 μM CTC at THB, washed three times for 2 min with THB, analysed and photographed under B2A filter of fluorescence microscope. Microphotographs were used to analyse distribution and amount of calcium ions by measurement of CTC fluorescence intensity with the Scn Image software.

Vacuoles were determined in the roots of seedlings which were untreated or treated for 72 h and for 7 days with kinetin under the white light microscope and under the florescence microscope after staining with AO mixture, respectively. Their membrane nature was confirmed under the phase-contrast microscope.

### DNA extraction and separation

DNA from the 2-cm-long apical part of roots of the untreated and 72-h kinetin-treated seedlings without meristems were extracted on ice with 2 % SDS, 0.5 M NaCl, 100 mM Tris–HCl and 50 mM EDTA at pH 8.0. DNA isolation and electrophoresis after RNA digestion with RNase A were carried out at 100 V for 3 h on a 1.5 % (*w*/*v*) agarose gel with 0.50 μg^.^ml^−1^ ethidium bromide according to Byczkowska et al. (2012).

## Results

### Detection of cell death and estimation number of dying and dead cells induced by kinetin

Double-coloured staining of nuclei with AO and EB based on their diverse abilities to permeate via cell membrane allowed the detection of cell death because AO permeated intact cells and emitted green fluorescence as a result of intercalation in the double-stranded DNA, while increasing changes in cell membrane induced by kinetin allowed EB to intercalate into nuclear DNA and gradually, by red colour, EB masked green colour of AO. Thus the computerised measurement of increasing RFI values of both fluorochromes allowed to count the number of living, dying and dead cells.

**Fig. 2:**
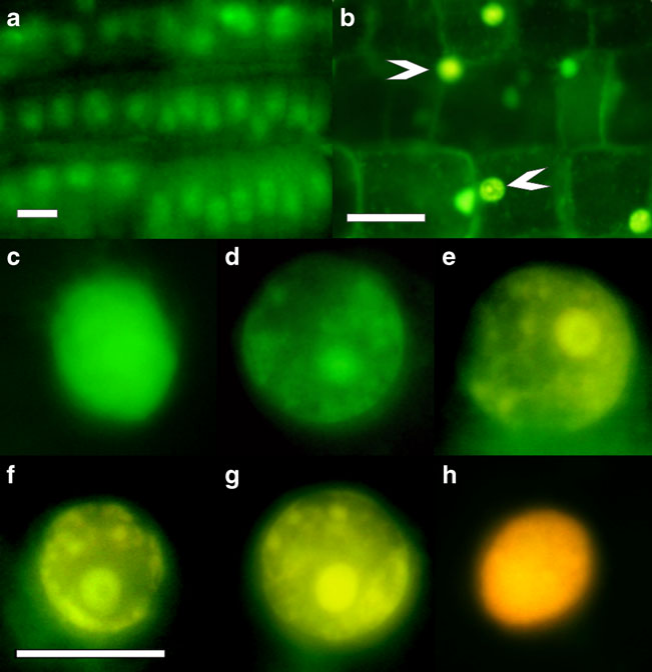
Microphotographs of the nuclei in living, dying and dead cells in the root cortex of *V*. *faba* ssp. *minor* seedlings after 46 μM kinetin treatment, detected by AO/EB staining in thin longitudinal sections of the zone I (**a**) and zone II (**b**) of root. **c** Green nuclei of living cells, green-yellow and green-yellow-orange nuclei of PCD cells with different forms of condensed (**d**, **e**) and disappearing (**f**, **g**) chromatin and bright red nuclei of dead cells (**h**). *Arrows* indicate PCD cells. *Scale bars* in **a**–**b** are 20 μm and in **c**–**h** are 10 μm

**Fig. 3:**
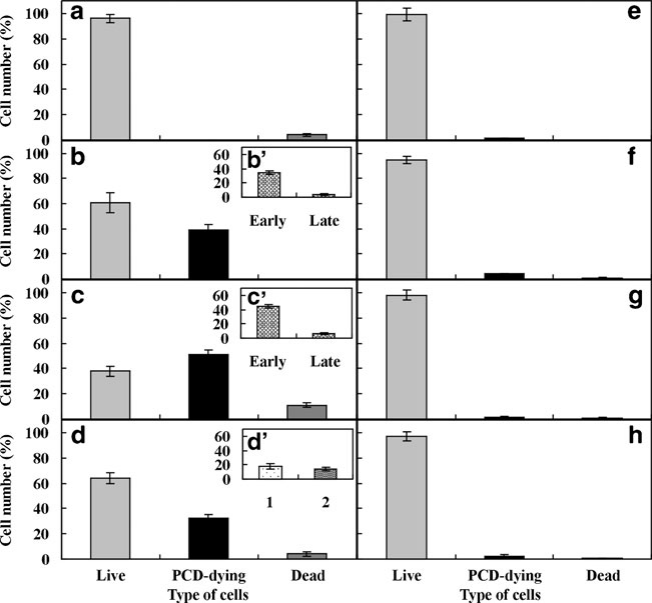
The number of living, PCD-dying and dead cells in the root cortex *V*. *faba* ssp. *minor* seedlings of untreated (**a**) and treated with kinetin for 48, 72, 96 h (**b**–**d**) as well as of dying cells at early and late stage of PCD (*b*’–*d*’) and those treated for 72 h with adenine (**e**), adenine with kinetin (**f**), mannitol (**g**) and mannitol with kinetin (**h**). *Error bars* represent the SE of the mean of three independent experiments

The results showed that kinetin induced cell death only in a mid cortex of parenchyma cells of the zone II (Fig. 1b; Fig. 2b) but not in the meristem cells of the zone I of the roots (Figs. 1a, 2a). In the root zone I of untreated or kinetin-treated plants for 48, 72 and 96 h, cells were alive (Fig. 2a) with unchanged green nuclei (Fig. 2c). In the zone II of roots of untreated plants after 72- and 96-h culture, some of cortex cells were dead; however, their number did not exceed about 4 % at 72 h (Fig. 3a). Kinetin treatment induced cell death in root zone II. Dying cells accounted for 40 % (Fig. 3b) and 51 % (Fig. 3c) of the total number of cells after 48- and 72-h exposition, respectively, and most of them (about 35 and 45 %, respectively) were at the early stage of PCD (Fig. 3 (b’, c’)) with green–yellow and partly condensed nuclear chromatin (Fig. 2d, e). The rest of them (about 5 and 6 %, respectively) were dark yellow and bright orange with degraded nuclear chromatin (Fig. 2 f, g) indicating that they were at the late stage of PCD (Fig. 3 (b’, c’)). After 96-h treatment with kinetin, the number of dying cells in root cortex lowered to 32 % of the total cell number (Fig. 3d). About 55 % of them were at the early while 45 % were at the late stage of PCD (Fig. 3 (d’)). After 72-h treatment, dead cells appeared. Their number was 11 %, but after 96-h treatment, it decreased to about 4 % (Fig. 3c, d). The nuclei of these cells had structurally normal dark orange or bright red nuclear chromatin (Fig. 2h). The differences between numbers of indicated cells were statistically significant (0.01 < *p* < 0.05).

### Effect of kinetin on morphology of nuclei and nuclear chromatin and DNA degradation during death

**Fig. 4:**
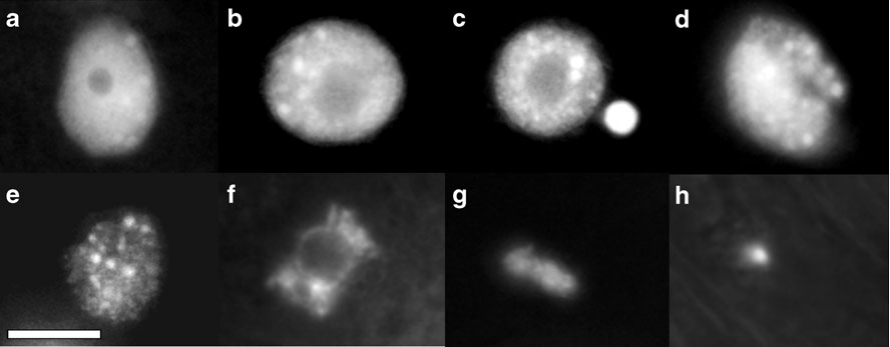
Microphotographs of the nuclei of kinetin-treated *V*. *faba* ssp. *minor* seedling root cells after DAPI showing the unchanged nuclei (**a**), nuclei with chromatin condensation (**b**), formation of micronuclei (**c**), nuclei invagination (**d**), disappearance of chromatin (**e**, **f**) and DNA fragmentation (**g**, **h**). *Scale bar* in “**e**” = 10 μm is applied to all figures

The AO/EB as well as DAPI staining allowed the observation of morphological changes in nuclei and nuclear chromatin after 48-, 72- and 96-h treatment with kinetin. The normal structure of nuclei of living cells (Figs. 2c, 4a) changed during kinetin-induced cell death. The results showed increasing condensation of nuclear chromatin (Fig. 2d–g, 4b), formation of micronuclei (Fig. 4c), invagination (Fig. 4d), degradation of chromatin (Fig. 4e) and nuclei fragmentation (Fig. 4g). The 96-h treatment with kinetin showed increasing nuclei degradation (Fig. 4f) and nuclei fragmentation (Fig. 4h).

**Fig. 5:**
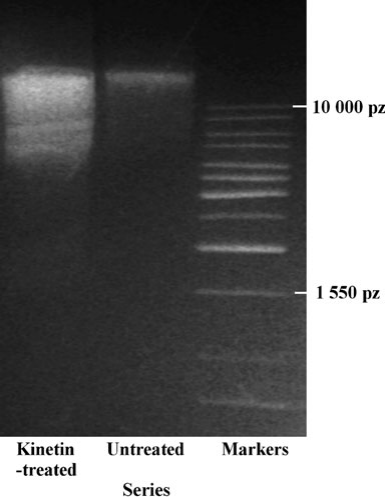
Electropherogram of samples of DNA isolated from the apical part of kinetin-treated and untreated *V*. *faba* ssp. *minor* seedling roots and DNA markers (1,550–10,000 bp)

However, the agarose gel electrophoresis of DNA extracted from the roots of seedlings treated for 72 h with kinetin showed that this degradation was not internucleosomal and only a “small smear” was visible (Fig. 5), while the profile area of the unornamented nuclei, in dying cells, increased by about 40 %.

### Kinetin changed the number of cells in particular phases of cell division cycle

**Fig. 6:**
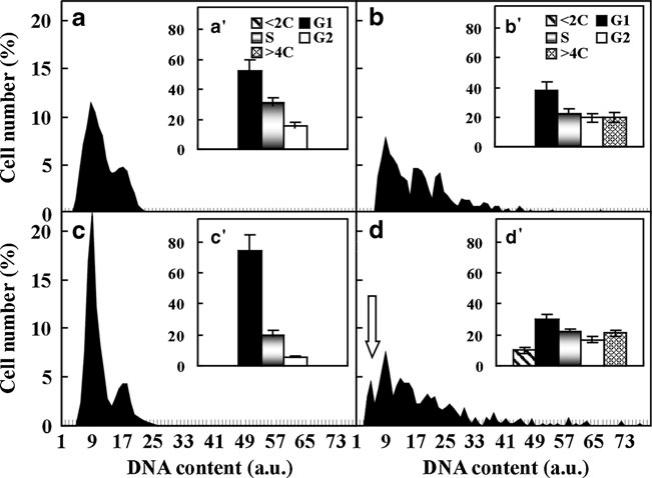
Histograms (**a**–**d**) displaying the percentage frequency distribution of cells (*a*’–*d*’) in the *G1*- (peak around 9 a.u.), *G2*- (peak around 19 a.u.) and *S*- (represented as the *gaps between the peaks* for phases G1 and G2), endoreplication- (>*4C* DNA) phases and hypoploid fraction (<*2C*) indicated by an *arrow*, microcytophotometrically determined in the zone I (**a**, *a*’, **c**, *c*’) and II (**b**, *b*’, **d**, *d*’) of untreated (**a**, *a*’, **b**, *b*’) and kinetin-treated (**c**, *c*’, **d**, *d*’) *V*. *faba* ssp. *minor* seedling roots. *Error bars* (*a*’–*d*’) represent the SE of the mean of three independent experiments

DAPI-microcytophotometric analysis allowed the estimation of DNA content for assigning cell nuclei to an appropriate phase of the cell cycle. It was shown that in the control plants cultured for 72 h in the zone I of roots, cells in the G1-, S- and G2-phase constituted about 53, 31 and 16 %, respectively (Fig. 6a (a’)), while the numbers of G1- and S-phase cells decreased by about 15 % and 9 %, respectively, and the number G2-phase cells increased by 4 % in the zone II (Fig. 6b (b’); *p* < 0.05). Moreover, there were approximately 20 % of endoreplicated cells with nuclei containing more than 4C DNA (Fig. 6b (b’)). The 72-h treatment with kinetin significantly affected DNA content in the seedling root cells. In the zone I, the number of G1-phase cells increased to about 75 %, the numbers of the other two types of cells decreased (Fig. 6c (c’); *p* < 0.01). In the zone II of the kinetin-treated roots, the number of G1-phase cells was lowered by about 45 % (*p* < 0.01) in comparison with the kinetin-treated zone I and by about 8 % (*p* < 0.05) in comparison with the zone II of untreated roots; the number of S-phase cells did not change while that of G2-phase cells decreased by about 3 % (Fig. 6d (d’); *p* < 0.05). The loss of G1- and G2-phase cells was replaced by 10 % of cells containing lower than 2 C DNA, forming the hypoploid fraction (Fig. 6d (d’)).

### Cellular dehydrogenase activity and conductivity during kinetin-induced cell death

**Fig. 7:**
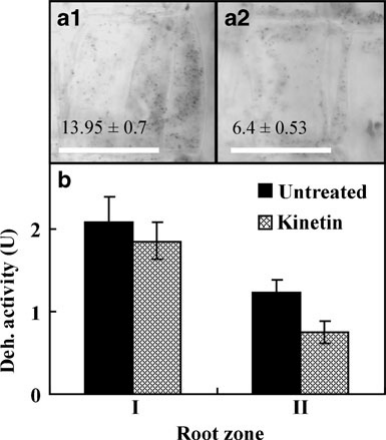
The number of mitochondria in the zone II of mid parts cortex cells of the untreated (*a1*) and kinetin-treated roots (*a2*) and activity of cellular dehydrogenases secreted (*b*) from the zone I and II of untreated and kinetin-treated *V*. *faba* ssp. *minor* seedling roots. *Scale bars* are 50 μm. *Error bars* represent the SE of the mean of three independent experiments

MTT determination is a useful colorimetric method to estimate the number of human, animal and fungal cells in suspensions (Mosmann 1983; Freimoser et al. 1999). According to this method, the number of viable cells has a linear relationship with absorbance of the MTT converted by dehydrogenase to formazan. The presented studies showed that the activity of dehydrogenases secreted from the zone I of roots of the 72-h kinetin-treated seedlings did not changed (*p* > 0.05); however, that in the zone II decreased to about 40 % (*p* < 0.01; Fig. 7a, b) in comparison to the control series. The activity of dehydrogenases after removal of kinetin from the 72-h culture was at same level (data not shown). The lowered level of dehydrogenase activity was accompanied with the significantly (*p* < 0.01) decreased (by about 54 %) number of mitochondria counted at the profile area of cortex cells from the root zone II of 72-h kinetin-treated seedlings (Fig. 7 (a1, a2)). Kinetin-induced cell death was also manifested by increment of conductivity of cell electrolytes secreted by the whole roots. At 72-h treated roots, its value increased from 11.5 ± 1.56 to 20.15 ± 3.25 μS (*p* < 0.01), while at 96-h treated roots, it decreased to 13.75 ± 2.20 μS (*p* < 0.05).

### Effect of adenine and mannitol on the number of kinetin-induced dying cells

**Fig. 8:**
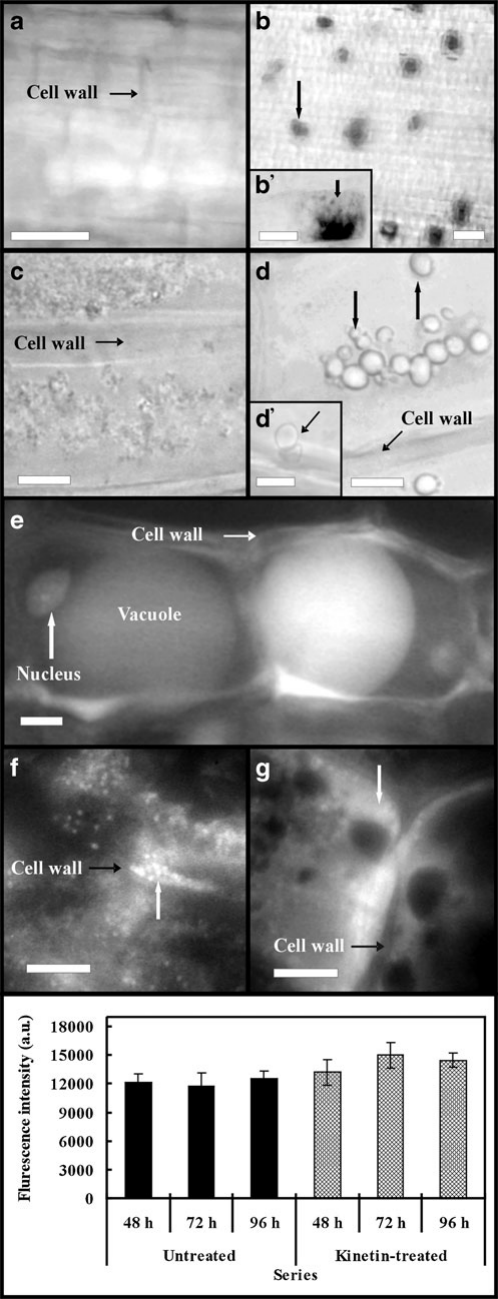
Microphotographs of ROS production (**a**, **b**, *b*’; *dark arrows*), formation of small (**c**, **d**, *d*’; *dark arrows*) and large (**e**) lytic vacuoles, calcium ion distribution (**f**, **g**, *white arrows*) and its amount (**h**) expressed in arbitrary units (a.u.) of fluorescence intensity of the zone II of untreated (**a**, **c**, **f**, **h**) and kinetin-treated (**b**, *b*’, **d**, *d*’, **e**, **g**, **h**) *V*. *faba* ssp. *minor* seedling roots. *Scale bar* in **a** is 100 μm, in **b** is 50 μm, in *b*’ and **c**, **d**, **e**, **f**, **g** is 10 μm and in *d*’ is 20 μm. *Error bars* in the “**i**” figure represent the SE of the mean of three independent experiments

Seedlings treated for 72 h with 50 μM adenine, the inhibitor of phosphoribosyl transferase, without or with kinetin showed that more than 95 % of cells of *V*. *faba* ssp. *minor* seedling roots were alive. Adenine induced death only in about 1 % of cells from the zone II of roots (Fig. 3c), while in the series with kinetin, about 4 % of cells were dying and about 1 % were dead in this zone (Fig. 3d). The differences between numbers of dying and dead cells in these series were not statistically significant (*p* > 0.05). Application of 50 μM mannitol induced cell death in about 2.5 % of cortex cells from the zone II of root of 72-h treated seedlings (Fig. 3e; 1.5 % were dying and 1 % were dead). Mannitol with kinetin increased the number of dying cells by about 1 % after 72-h treatment with kinetin (Fig. 3f). Their number was similar (1–2 %) after 48- and 96-h treatment with kinetin and mannitol. Differences between values were not statistically significant (*p* > 0.05). Moreover, mannitol inhibited ROS production in kinetin-treated roots (Fig. 8b (b’)) and the microscopic pictures of root cortex cells were similar to the untreated series of *V*. *faba* ssp. *minor* seedlings (Fig. 8a). The number of ROS-producing cells estimated per microscope area in the roots treated with kinetin and mannitol for 48 h was 1 %, then it increased to 10 % (72 h) and decreased to 2 % (96 h).

### Kinetin induced vacuole formation, increased the amount of cytosolic calcium ions, affected cell and root lengths as well as increased weight and width of roots

In the parenchyma cells of root cortex of *V*. *faba* ssp. *minor* seedlings in the kinetin-treated series under the white light microscope, the presence of vacuolar structures (Fig. 8d; not observed in the untreated series, Fig. 8c) with confirmed membrane nature by phase-contrast microscope (Fig. 8 (d’)) was shown. These vacuoles underwent fusion forming large vacuoles (Fig. e) with acid pH (green colour after AO staining) in root cells of seedlings treated with kinetin for 7 days.

The intensity of calcium ion CTC fluorescence staining in 48-, 72- and 96-h treated *V*. *faba* ssp. *minor* seedling root cells increased by 10, 30 and 14 %, respectively, in the levels of cytosolic calcium ions (Fig. 8i). However, only the 72-h treatment resulted in statistically significant changes (*p* < 0.05; Fig. 8i).

After 72-h incubation of *V*. *faba* ssp. *minor* seedlings with 46.0 μM kinetin, the average length of roots decreased by about 45 % from 5.31 ± 0.59 cm to 2.86 ± 0.41 (Fig. 1; *p* < 0.01), while the weight of 2-cm-long apical parts of roots increased from 56.9 ± 3.7 to about 100.2 ± 4.4 mg (*p* < 0.02). Width of the apical part of roots increased by about 40 % from 1.50 ± 0.09 to 2.1 ± 0.05 mm (*p* < 0.05). Moreover, the zone II cells diminished from 208.5 ± 25.5 μm to 135.4 ± 13.0 (*p* < 0.05).

## Discussion

Isoprenoid and aromatic N^6^-substituted adenine derivatives, endogenously occurring as free bases, nucleosides, nucleotides and glucosides, known as cytokinins, often present at very low concentrations, are important plant growth regulators and animal cell differentiation factors (Barciszewski et al. 2007; Doležal et al. 2007).

In plants, they promote cell division, callus growth, seed germination, redifferentiation into adventitious buds or roots, bud differentiation, branching, chlorophyll and starch production, resistance to plant pathogens, apical dominance and leaf senescence (Barciszewski et al. 2007; Doležal et al. 2007). In human and animals, cytokinins participate in the control of cell differentiation (Ishii et al. 2002; Berge et al. 2006). In cancer cells (Ishii et al. 2002; Cabello et al. 2009) as well as in plant cell-suspension cultures (Carimi et al. 2003; Mlejnek et al. 2003) and *in planta* systems (Carimi et al. 2003), various natural cytokinins can induce cell death which is manifested by cytoplasm and cell shrinkage (Mlejnek et al. 2003; Jan et al. 2008), nuclei condensation (Carimi et al. 2003; Gahan et al. 2003; Mlejnek et al. 2003) and fragmentation (Ishii et al. 2002; Kobori et al. 2007). Sometimes, chromatin breakdown into internucleosomal DNA fragments (Gahan et al. 2003; Carimi et al. 2003; Mlejnek et al. 2003), release of cytochrome c (Carimi et al. 2003), formation of DNA-containing, apoptotic-like bodies (Ishii et al. 2002), presence of apoptosis-specific caspase/metacaspase enzymes (van Doorn and Woltering 2005; Mlejnek and Procházka 2002; Parish and Li 2010) as well as activation of 28 kDa endonuclease are observed (Gahan et al. 2003).

The main aim of this research was to show that the kinetin-induced cell death in roots of *V*. *faba* ssp. *minor* seedlings is connected with changes of plasma, nuclear (Zhao et al. 2001) and mitochondrial (Carimi et al. 2003) membranes—the first and common symptoms of plant and animal PCD process (van Doorn and Woltering 2005; Cabello et al. 2009), during which mitochondria and then nucleus tend to be the last organelles to be degraded during the execution phase of PCD (van Doorn and Woltering 2005; Cacas 2010). All this allowed to apply the unequivocal unique AO/EB fluorescence staining method to detect PCD (Byczkowska et al. 2012) and to show changes in nuclei which are the unquestionable hallmarks of this process in animals (Ribble et al. 2005) and in plants (van Doorn 2011; van Doorn et al. 2011). This method due to EB ability to penetrate via cellular membrane enables the detection of both the first and the last steps of cell death, making it a very universal method for cell death evaluation (Byczkowska et al. 2012). Using this method, it was shown that kinetin induced PCD process only in the root cortex parenchyma cells but not in the root meristem cells. The number of dying cells increased after 48- and 72-h treatment with kinetin, and after 96 h, their number decreased.

The fact that kinetin induced death in the cortex cells was also evidenced by the number of cells in particular phases of the cell cycle. It was shown that in the zone I of roots, where the dying or dead cells were not detected, kinetin arrested cells at G1 phase whereas in the zone II of roots, kinetin decreased the number of G1- and G2-phase cells and the fraction of hypoploid nuclei appeared, where the G2- and G1-phase cells, after progressive degradation, could be shifted. These results clearly suggesting that the G1- and G2-phase cells of the zone II of *V*. *faba* ssp. *minor* roots were directed by kinetin to PCD were in agreement with those presented for animal cells which undergo apoptosis after cytokinin treatment (Barciszewski et al. 2007). Cytokinins regulating cyclin-dependent kinase activities control cell proliferation of many tumour cell lines arresting it at G1/S and/or G2/M transition points triggering apoptosis (Havlíček et al. 1997). The block of cell proliferation and PCD induction was also detected in carrot (*D*. *carota* L.) and in *A*. *thaliana* cell cultures after treatment with BAP (Carimi et al. 2003).

PCD is also manifested by increment of conductivity of culture media, resulting from the changes of cell membranes potential leading to leakage of cell electrolytes (Kawai-Yamada et al. 2004; Palavan-Unsal et al. 2005). In the present research, kinetin induced up to two-fold increase in cell electrolyte leakage. Both removal of kinetin from the culture media (data not shown) and 96-h treatment with kinetin reduced cell electrolyte leakage. Kinetin also decreased by about 40 % cellular dehydrogenase activities released from cells of the zone II of roots, unchanging activities of cellular dehydrogenases released from the zone I of roots. This study showed that the measurement of total cellular dehydrogenases activity, which is usually used to determine the number of animal viable cells (Mosmann 1983; Freimoser et al. 1999), might be also applied to *in planta* systems, where the number of living cells might be expressed as a percentage of dehydrogenases activity. These values (about 40 %) correlated with similar percentage (about 40 %) of alive root cortex cells and were also similar to the number of mitochondria (about 45 %) remaining in the root cortex cells of *V*. *faba* ssp. *minor* after 72-h treatment with kinetin. Their number may describe a minimum number of functional mitochondria designated as the “point of no return” (van Doorn 2005), indicating that these cells are protected against kinetin-induced death.

Besides the above, some other values of PCD hallmarks induced after 72-h treatment with kinetin oscillated around 40 %, namely decrease in root and cell length, increment in weight and width of 2-cm-long apical parts of roots as well as increase in nuclear profile area. All these findings suggested that kinetin-induced cell death process was specific and expressed itself with similar intensity on both morphological and metabolic levels.

Being in agreement with the point of view of van Doorn et al. (2011) which indicates that apoptosis is not present in plants, our results showed that kinetin, the animal and plant cell growth and differentiation regulator (Barciszewski et al. 2007), in the root cortex cells of *V*. *faba* ssp. *minor* seedlings induced programmed cell death. It was characterised by (1) formation of lytic vacuoles (small vacuoles were also typical of animal necrosis; McCall 2010) increased by fusion (typical of vacuolar type of death, van Doorn 2011), (2) nuclear envelope disassembly, (3) nuclear segmentation, (4) nuclear chromatin condensation (also typical of animal apoptosis, van Doorn 2011, and in ACC-induced PCD in *V*. *faba* ssp. *minor* seedlings, Byczkowska et al. 2012), (5) increase and then decrease in cell electrolyte leakage (Kawai-Yamada et al. 2004; Palavan-Unsal et al. 2005), (6) swelling and degradation of nuclei indicated vacuolar type of cell death (Jan et al. 2008; Scott and Logan 2008; van Doorn 2011; van Doorn et al. 2011) and (7) inhibition of longitudinal growth of cells leading to decreased length and increased width of roots. However, kinetin-induced PCD effects including increase in (8) cytosolic calcium ions and (9) ROS production (also hallmarks of animal necrotic or plant non-autolytic type of cell death; Cacas 2010; Collazo et al. 2006; Jan et al. 2008; van Doorn 2011; van Doorn et al. 2011) “it does not automatically mean that the example is to be classified as a necrotic PCD” (van Doorn 2011). Decrease in the number of dying cells after 96-h treatment with kinetin accompanied with decrease in conductivity, amount of cytosolic calcium ions and ROS producing cells strongly indicated that this process was transient. ROS production, being of mitochondrial and/or cellular origin (Mlejnek et al. 2003), is connected with the loss of mitochondrial membrane potential which was caused by depletion of mitochondrial ATP levels leading to the oxidative DNA lesions followed by DNA fragmentation (Roy et al. 2008). DNA degradation in *V*. *faba* ssp. *minor* roots might be also induced by ROS or by calcium ions (Jan et al. 2008), ubiquitous signal molecules, that are involved in the regulation of almost all cellular functions (Bergner and Huber 2008), whose cytosolic concentration increased during kinetin-induced cell death. Endoplasmic reticulum (ER) is the main intracellular Ca^2+^ reservoir (Bergner and Huber 2008), and when membrane of ER loses its potential and/or its integrity, during PCD, it is released. These ions might be rapidly taken up by mitochondria, rendering cells less responsive to death stimuli (Cacas 2010) and this also might induced specific nucleus-containing nucleases (NUC 1, DNaseI and DNaseII) which led to DNA condensation, fragmentation and marginalisation (Jan et al. 2008). Chromatin condensation as well as micronuclei formation and loss of cell membrane integrity (van Doorn 2011; van Doorn et al. 2011) are the hallmarks of apoptotic animal cell death (Scott and Logan 2008); however, they resemble PCD induced in plants by BAP, another natural plant cytokinin (Carimi et al. 2003; Gahan et al. 2003; Mlejnek et al. 2003; Barciszewski et al. 2007). However, in the kinetin-treated roots, internucleosomal DNA degradation was not visible, similarly as after ACC application in *V*. *faba* ssp. *minor* roots during first steps of aerenchyma formation (Byczkowska et al. 2012). Kinetin-induced cell death also led to aerenchyma formation (data not shown) but did not cause the elimination of all cortex cells from the root and whole roots and/or whole of *V*. *faba* ssp. *minor* seedlings. This was supported by decrease in the number of dying cells after 96-h treatment with kinetin. Moreover, about 4 % (after 48 h), 6 % (after 72 h) and 15 % (after 96 h) of cells were directed to the late-degradation-PCD stage, suggesting protection of some cells by eliminating others. This fact can be also explained by the lack of DNA ladder which is observed during cell death but mainly in cell cultures e.g. after treatment with BAP (Carimi et al. 2003).

All of these results, as well as the fact that aerenchyma formation is a vacuolar type of PCD (van Doorn 2011; van Doorn et al. 2011), indicate that cell death induced in *V*. *faba* ssp. *minor* seedling roots is the kinetin-specific vacuolar type of death.

Analyses of hallmarks fundamental for PCD (Jan et al. 2008; Cacas 2010; van Doorn 2011; van Doorn et al. 2011) showing the nature of kinetin-induced cell death also allowed to propose a probable mechanism of its induction. It seems that kinetin is converted with phosphoribosyl transferase to corresponding monophosphates (Mlejnek and Doležel 2005), purine ligands specific for histidine kinases receptors (AHK2, AHK3 and AHK) discovered in ER membrane of *Arabidopsis* and *Zea mays* (Caesar et al. 2011; Doležal et al. 2007; Barciszewski et al. 2007). Then, monophosphates binding to receptors induce efflux of calcium ions from ER (Bergner and Huber 2008) and directly or indirectly arrest cells in the G1 phase and G2 phase and direct some of them onto the path of programmed death with DNA condensation, nuclei segmentation and DNA degradation by nucleases (Jan et al. 2008; van Doorn et al. 2011). Simultaneously, calcium ions influx to mitochondria induces ROS production (Cacas 2010; Collazo et al. 2006; Jan et al. 2008; van Doorn et al. 2011), which independently could destroy nuclear DNA (Roy et al. 2008) and/or signal specific cell death-dependent processes. Finally, gradually increasing by fusion, acidic lytic vacuoles (McCall 2010; Scott and Logan 2008; van Doorn et al. 2011) engulf and destroy protoplasts (Jan et al. 2008; Scott and Logan 2008; van Doorn et al. 2011) of dying cells.

## References

[CR1] Barciszewski J, Massino F, Clark BFC (2007). Kinetin—a multiactive molecule. Int J Biol Macromol.

[CR2] Berge U, Kristensen P, Rattan SIS (2006). Kinetin-induced differentiation of normal human keratinocytes undergoing aging in vitro. Ann NY Acad Sci.

[CR3] Bergner A, Huber RM (2008). Regulation of the endoplasmic reticulum Ca^2+^-store in cancer. Anti-cancer Agents Med Chem.

[CR4] Byczkowska A, Kunikowska A, Kaźmierczak A (2012) Determination of ACC-induced cell programmed death in roots of *Vicia faba* ssp. *mino*r seedlings by acridine orange and ethidium bromide staining. Protoplasma. doi:10.1007/s00709-012-0383-9 10.1007/s00709-012-0383-9 PMC3557382 22350735

[CR5] Cabello CM, Bair WB, Ley S, Lamore SD, Azimian S, Wondrak GT (2009). The experimental chemotherapeutic N6-furfuryladenosine (kinetin-riboside) induces rapid ATP depletion, genotoxic stress, and CDKN1A (p21) upregulation in human cancer cell lines. Biochem Pharmacol.

[CR6] Cacas JL (2010). Devil inside: does plant programmed cell death involve the endomembrane system?. Plant Cell Environ.

[CR7] Caesar K, Thamm AMK, Witthöft J, Elgass K, Huppenberger P, Grefen C, Horak J, Harter K (2011). Evidence for the localization of the Arabidopsis cytokinin receptors AHK3 and AHK4 in the endoplasmic reticulum. J Exp Botany.

[CR8] Carimi F, Zottini M, Formentin E, Terzi M, Lo SF (2003). Cytokinins: new apoptotic inducers in plants. Planta.

[CR9] Choi BH, Kim W, Wang QC, Kim DC, Tan SN, Yong JWH, Kim KT, Yoon HS (2008). Kinetin riboside preferentially induces apoptosis by modulating Bcl-2 family proteins and caspase-3 in cancer cells. Cancer Lett.

[CR10] Collazo C, Chacon O, Boras O (2006). Programmed cell death in plants resembles apoptosis of animals. Biotecnol Aplic.

[CR11] Delaval B, Birnbaum D (2007). A cell cycle hypothesis of cooperative oncogenesis. Int Journal Oncol.

[CR12] Doležal K, Popa I, Hauserová E, Spíchal L, Chakrabarty K, Novák O, Kryštof V, Voller J, Holub J, Strnad M (2007). Preparation, biological activity and endogenous occurrence of N6-benzyladenosines. Bioorg Med Chem.

[CR13] Freimoser FM, Jakob CA, Aebi M, Tuor U (1999). The MTT [3-(4,5-dimethylthiazol-2-yl)-2,5-diphenyltetrazolium bromide] assay is a fast and reliable method for colorimetric determination of fungal cell densities. Appl Environ Microbiol.

[CR14] Gahan PB, Wang L, Bowen ID, Winters C (2003). Cytokinin-induced apoptotic nuclear changes in cotyledons of *Solanum aviculare* and *Lycopersicon esculentum*. Plant Cell Tiss Organ Cult.

[CR15] Griffaut B, Bos R, Maurizis JC, Madelmont JC, Ledoigt G (2004). Cytotoxic effects of kinetin riboside on mouse, human and plant tumour cells. Int J Biol Macromol.

[CR16] Havlíček L, Hanuš J, Veselý J, Leclerc S, Meijer L, Shaw G, Strnad M (1997). Cytokinin-derived cyclin-dependent kinase inhibitors: synthesis and cdc2 inhibitory activity of olomoucine and related compounds. J Med Chem.

[CR17] Hotz MA, Traganos F, Darzynkiewicz Z (1992). Changes in nuclear chromatin related to apoptosis or necrosis induced by the DNA topoisomerase II inhibitor fostriecin in MOLT-4 and HL-60 cells are revealed by altered DNA sensitivity to denaturation. Exp Cell Res.

[CR18] Hübner B, Strickfaden H, Müller S, Cremer M, Cremer T (2009). Chromosome shattering: a mitotic catastrophe due to chromosome condensation failure. Eur Biophys J.

[CR19] Ishii Y, Hori Y, Sakai S, Honma Y (2002). Control of differentiation and apoptosis of human myeloid leukemia cells by cytokinins and cytokinin nucleosides, plant redifferentiation-inducing hormones. Cell Growth Differ.

[CR20] Jan N, Hussain M, Andrabi KI (2008). Programmed cell death or apoptosis: do animals and plants share anything in common. Biotechnol Mol Biol Rev.

[CR21] Jennings DB, Ehrenshaft M, Mason Pharr D, Williamson JD (1998). Roles for mannitol and mannitol dehydrogenase in active oxygen-mediated plant defense. Plant Biol.

[CR22] Kawai-Yamada M, Ohori Y, Uchimiya H (2004). Dissection of Arabidopsis Bax inhibitor-1 suppressing Bax-, hydrogen peroxide-, and salicylic acid-induced cell death. Plant Cell.

[CR23] Kaźmierczak A (2010). Endoreplication in *Anemia phyllitidis* coincides with the development of gametophytes and male sex. Physiol Plant.

[CR24] Kobori S, Masuda Y, Horii M, Marubashi W (2007). High levels of the cytokinin BAP suppress programmed cell death in hybrid tobacco cells (*Nicotiana suaveolens* × *N*. *tabacum*) expressing hybrid lethality. Plant Biotechnol.

[CR25] McCall K (2010). Genetic control of necrosis—another type of programmed cell death. Curr Op Cell Biol.

[CR26] Mlejnek P, Doležel P (2005). Apoptosis induced by N6-substituted derivatives of adenosine is related to intracellular accumulation of corresponding mononucleotides in HL-60 cells. Tox in Vitro.

[CR27] Mlejnek P, Procházka S (2002). Activation of caspase-like proteases and induction of apoptosis by isopentenyladenosine in tobacco BY-2 cells. Planta.

[CR28] Mlejnek P, Doležel P, Procházka S (2003). Intracellular phosphorylation of benzyladenosine is related to apoptosis induction in tobacco BY-2 cells. Plant Cell Environ.

[CR29] Mosmann T (1983). Rapid colorimetric assay for cellular growth and survival: application to proliferation and cytotoxicity assays. J Immunol Methods.

[CR30] Palavan-Unsal N, Buyuktuncer ED, Tufekci MA (2005). Programmed cell death in plants. J Cell Mol Biol.

[CR31] Parish RW, Li SF (2010). Death of a tapetum: a programme of developmental altruism. Plant Sci.

[CR32] Rao GV, Kumar S, Islam M (2008). Mansour SE (2008) Folk medicines for anticancer therapy—a current status. Cancer Ther.

[CR33] Ribble D, Goldstein NB, Norris DA, Shellman YG (2005). A simple technique for quantifying apoptosis in 96-well plates. BMC Biotechno l.

[CR34] Roy A, Ganguly A, Dasgupta SB, Das BB, Pal C, Jaisankar P, Majumder HK (2008). Mitochondria dependent ROS-mediated programmed cell death (PCD) induced by 3,3'-diindolylmethane (DIM) through Inhibition of FoF1-ATP synthase in unicellular protozoan parasite *Leishmania donovani*. Mol Pharm.

[CR35] Scott I, Logan DC (2008). Mitochondria and cell death pathways in plants. Plant Signal Behav.

[CR36] Shemarova IV (2010). Signaling mechanisms of apoptosis-like programmed cell death in unicellular eukaryotes. Comp Biochem Physiol B Biochem Mol Biol.

[CR37] Taraphdar AK, Roy M, Bhattacharya RK (2001). Natural products as inducers of apoptosis: implication for cancer therapy and prevention. Curr Sci.

[CR38] Van Doorn WG (2005). Plant programmed cell death and the point of no return. Trends Plant Sci.

[CR39] van Doorn WG (2011). Classes of programmed cell death in plants, compared to those in animals. J Exp Bot.

[CR40] van Doorn WG, Woltering EJ (2005). Many ways to exit? Cell death categories in plants. Trends Plant Sci.

[CR41] van Doorn WG, Beers EP, Dangl JL, Franklin-Tong VE, Gallois P, Hara-Nishimura I, Jones AM, Kawai-Yamada M, Lam E, Mundy J, Mur LAJ, Petersen M, Smertenko A, Taliansky M, Van Breusegem F, Wolpert T, Woltering E, Zhivotovsky B, Bozhkov PV (2011). Morphological classification of plant cell deaths. Cell Death Differ.

[CR42] Zhao Y, Wu M, Shen Y, Zhai Z (2001). Analysis of nuclear apoptotic process in a cell-free system. Cell Mol Life Sci.

